# Overexpression of IκB⍺ modulates NF-κB activation of inflammatory target gene expression

**DOI:** 10.3389/fmolb.2023.1187187

**Published:** 2023-05-09

**Authors:** Polly Downton, James S. Bagnall, Hazel England, David G. Spiller, Neil E. Humphreys, Dean A. Jackson, Pawel Paszek, Michael R. H. White, Antony D. Adamson

**Affiliations:** ^1^ Faculty of Biology, Medicine and Health, University of Manchester, Manchester, United Kingdom; ^2^ Genome Editing Unit, Faculty of Biology, Medicine and Health, University of Manchester, Manchester, United Kingdom

**Keywords:** NF-κB, inflammation, IκB⍺, overexpression, gene expression, localisation

## Abstract

Cells respond to inflammatory stimuli such as cytokines by activation of the nuclear factor-κB (NF-κB) signalling pathway, resulting in oscillatory translocation of the transcription factor p65 between nucleus and cytoplasm in some cell types. We investigate the relationship between p65 and inhibitor-κB⍺ (IκBα) protein levels and dynamic properties of the system, and how this interaction impacts on the expression of key inflammatory genes. Using bacterial artificial chromosomes, we developed new cell models of IκB⍺-eGFP protein overexpression in a pseudo-native genomic context. We find that cells with high levels of the negative regulator IκBα remain responsive to inflammatory stimuli and maintain dynamics for both p65 and IκBα. In contrast, canonical target gene expression is dramatically reduced by overexpression of IκBα, but can be partially rescued by overexpression of p65. Treatment with leptomycin B to promote nuclear accumulation of IκB⍺ also suppresses canonical target gene expression, suggesting a mechanism in which nuclear IκB⍺ accumulation prevents productive p65 interaction with promoter binding sites. This causes reduced target promoter binding and gene transcription, which we validate by chromatin immunoprecipitation and in primary cells. Overall, we show how inflammatory gene transcription is modulated by the expression levels of both IκB⍺ and p65. This results in an anti-inflammatory effect on transcription, demonstrating a broad mechanism to modulate the strength of inflammatory response.

## Introduction

The nuclear factor kappa B (NF-κB) signalling pathway is involved in the regulation of a wide range of cellular processes. NF-κB signalling is a key mediator of the immune system, and is activated in many cell types in response to viral and bacterial pathogens and cytokine signalling cascades (Hayden and Ghosh, 2008). Dysregulated NF-κB signalling is linked to cancer, inflammatory and autoimmune diseases (Perkins, 2012; Taniguchi and Karin, 2018; Barnabei et al., 2021).

In mammals there are five NF-κB proteins; p65/RelA, RelB, cRel, p50, and p52, which hetero- and homo-dimerise in several possible combinations (Hayden and Ghosh, 2008). NF-κB transcription factor complexes (canonically, RelA/p65:p50) are found in the cytoplasm of resting cells bound to inhibitor κB (IκB) family molecules, the most abundant of which is IκB⍺. Stimulation of cells with a pro-inflammatory signal, such as tumour necrosis factor α (TNF⍺), leads to a signalling cascade which results in IκB kinase (IKK) activation, and phosphorylation of both p65 and IκB⍺. The phosphorylated IκB⍺ is subsequently ubiquitinated and targeted for proteasomal degradation, releasing p65 to translocate to the nucleus and bind to target gene promoters, including IκB⍺. Newly synthesised IκB⍺ can translocate to the nucleus, where it may bind p65 to result in relocation of the p65:IκB⍺ complex to the cytoplasm.

We and others have shown that p65 oscillates between nucleus and cytoplasm in the presence of continued stimulation (Nelson et al., 2004; Sung et al., 2009; Tay et al., 2010). This oscillatory behaviour quickly becomes asynchronous in a population of cells following initial stimulation, meaning single cell analyses are essential for detection and quantification (Paszek et al., 2010; Aqdas and Sung, 2022). Exogenous plasmid and lentiviral systems typically include a constitutive promoter to drive coding sequence expression, which can result in perturbation of transcript copy number and protein expression level. It has been shown that overexpression of p65 results in downstream effects on target gene expression (Sung et al., 2009; Lee et al., 2014), often resulting in increased activation of pro-inflammatory target genes.

We previously generated a clonal cell line containing a stably-integrated recombinant Bacterial Artificial Chromosome (BAC) expressing IκB⍺ fused to eGFP, (Adamson et al., 2016). This system of BAC mediated overexpression provides copy number perturbation and increased levels of protein, but has the advantage of maintaining regulation of the transgene in a pseudo-genomic context. We showed that the oscillatory timing of IκB⍺ expression is robust and out-of-phase with p65 nuclear:cytoplasmic (N:C) translocation. We identified a heterogeneous refractory period of response to pulsatile cytokine treatment, predicted to be controlled by a post-translational switch between the ligand activated receptor and the IKK, which determines whether cells can respond to a second pulse of cytokine stimulation.

The delivery of the IκB⍺-eGFP BAC to cells and successful stable integration into genomic DNA resulted in overexpression of IκB⍺. Previous observations have shown that p65 protein level has an effect on NF-κB target gene expression (Sung et al., 2009). We investigate the effect of IκB⍺ feedback on oscillatory behaviour and downstream gene expression using an overexpression system. We find, using BAC stable cell lines and cells derived from transgenic mice, that although oscillatory period is robust to differing expression levels, IκB⍺ overexpression results in potent downregulation of the transcription of canonical NF-κB target genes. We provide evidence of a mechanism relating this to increased levels of free IκB⍺ in the nucleus, where IκB⍺ directly competes for translocated p65.

## Materials and methods

### Reagents and cell culture

SK-N-AS neuroblastoma (Cat. No. 94092302) cells were obtained from the European Collection of Authenticated Cell Cultures (ECACC). Cells were cultured according to ECACC protocols, and frozen down to form a low passage working stock. Working stocks were screened to ensure the absence of *Mycoplasma* every 3 months using LookOut *Mycoplasma* PCR Detection Kit (Cat. No. D9307 Sigma, United Kingdom). Cells were cultured in Modified Eagle’s Medium supplemented with 10% foetal bovine serum (FBS) and 1% non-essential amino acids. Treatments used recombinant human TNF⍺ (10 ng/mL; Merck 654205) and/or LMB (20 ng/mL, Merck 431050).

Primary mouse fibroblasts were cultured from ear tissue biopsies. Tissue was minced and cells isolated by incubation in collagenase (Sigma C2674) for 30 min at 37°C. Cells were maintained in Dulbecco’s Modified Eagle’s Medium supplemented with 10% FBS and penicillin/streptomycin. Cells were treated with recombinant mouse TNF⍺ (10 ng/mL, Merck 654245).

### IκB⍺-eGFP construct

We have previously described the IκB⍺-eGFP recombinant BAC (Adamson et al., 2016). Briefly, using a GalK selection/counterselection recombineering strategy (Warming et al., 2005) we seamlessly integrated the fluorescent protein gene in place of the STOP codon, to create a C-terminally tagged fusion construct that is expressed in a pseudo-genomic context when transfected/integrated into cells. This construct is available on request.

### Generation of IκB⍺-eGFP clonal cell lines

BAC DNA for transfection was prepared using the BAC100 Nucleobond kit (Macherey-Nagel, Germany). Cells were transfected using ExGen500 transfection reagent (Fermentas, United Kingdom) and clonal cell lines were derived by cell sorting as previously described (Adamson et al., 2016). Cell lines are available on request.

### Lentiviral packaging and transduction

Lentiviral constructs were cloned and lentivirus produced as previously described (Bagnall et al., 2015). Clonal cell lines were transduced with lentivirus encoding a human p65-mCherry C-terminal fusion protein under control of the ubiquitin ligase C promoter.

### qRT-PCR

For RNA isolation, cells were seeded into 6-well plates (100000/well). After treatments as described, cells were washed once with cold PBS then lysed. Total RNA was isolated using the High Pure RNA isolation kit (Roche). RNA concentration was determined using a Nanodrop ND-1000 spectrophotometer (Thermo). RNA was reverse transcribed to cDNA using the SuperScript VILO cDNA synthesis kit (Life Technologies). The resulting cDNA was analysed by qRT-PCR on a LightCycler 480 using SYBR Green 1 Master Mix (Roche). Relative fold change in expression was determined by the ddCt method, using *PPIA* expression as a housekeeping control. Primer sequences are given in Supplementary Table S1.

### smRNA-FISH

Custom smRNA-FISH probe sets were designed against coding sequences, and UTRs when necessary, using the Stellaris FISH Probe Designer (Biosearch Technologies Inc.). Probes were conjugated with Quasar 570 or Quasar 670. Sequences are given in Supplementary Table S2. *eGFP* transcripts were detected using the pre-designed Quasar 570 probe set (Biosearch Technologies Inc., VSMF-1014-5).

Cells were seeded into 12-well plates (40000/well) containing coverslips pre-coated with poly-L-lysine. Following treatment, cells were washed, fixed with 3.7% formaldehyde in PBS for 10 min, then permeabilised with 70% ethanol for 2–24 h at 4°C. Coverslips were washed (10% formamide in 2 X SSC) then hybridised with probe mix (probe/s of interest in 10% formamide in 2 X SSC containing 100 mg/mL dextran sulphate) overnight at 37°C. Coverslips were washed, incubated with DAPI, then mounted in Vectashield for imaging.

Images were acquired on a DeltaVision (Applied Precision) microscope using a 60x NA 1.42 Plan Apo N objective and a Sedat Quad filter set. The images were collected using a CoolSNAP HQ (Photometrics) camera with z optical spacing of 0.2 μm. Raw images were deconvolved using softWoRx software. Deconvolved image stacks were analysed using FISH-quant to determine transcript numbers per cell (Mueller et al., 2013). Exemplar maximum projection images were generated in Fiji (Schindelin et al., 2012).

### Western blotting

Cells were seeded in 35 mm dishes (50000/dish) two days prior to sample collection. Samples were washed once with cold PBS, then lysed in hot buffer (1% (w/v) SDS, 10% (v/v) glycerol, 10% (v/v) β-ME, 40 mM Tris pH 6.8, 0.01% (w/v) bromophenol blue). Proteins were resolved on polyacrylamide gels run under denaturing conditions, then transferred to nitrocellulose membrane (Protran BA-83, GE Healthcare). Membranes were blocked with 5% (w/v) skim milk powder in TBS-T prior to overnight incubation with primary antibody (anti-IκB⍺, Cell Signalling Technology #9242, 1:1,000; anti-ICAM1, Santa Cruz Biotechnology sc-8439, 1:200; anti-⍺-Tubulin, Sigma Aldrich T6199, 1:4,000; anti-Vinculin, Cell Signalling Technology 4650S, 1:1,000). Membranes were washed with TBS-T, incubated with HRP-conjugated secondary antibody (CST #7074 or #7076, 1:1,000), washed and developed using Luminata Crescendo substrate (Millipore WBLUR0500). Signal was detected using Carestream Kodak BioMax MR film (Sigma-Aldrich). Unprocessed images of films are provided in Supplementary Figure S7. Signal was quantified using Fiji (Schindelin et al., 2012).

### Confocal time lapse imaging

Cells were seeded into 35 mm glass-bottomed dishes (Greiner) and imaged using several Zeiss confocal microscopes (LSM Pascal, Exciter, 710, 780, 880) with Fluar 40x NA 1.3 objectives. Nuclei were stained with Hoechst 33342 prior to imaging where indicated (Thermo Fisher H3570, incubation with 100 ng/mL for 20 min). Cells were maintained at 37°C in humidified 5% CO_2_ throughout image acquisition. Image capture used Zeiss software (Aim version 4.2, Zen 2010b SP1 or Zen 2.1 SP3 FP2). Quantification of IκB⍺-eGFP fluorescent signal of whole cells was performed using region of interest (ROI) analysis in Zen 2010b SP1 software. Normalised expression level was calculated relative to average cell fluorescence intensity prior to treatment. Quantification of dynamic parameters was performed by a custom script based on the ‘findpeaks’ function using Matlab 2020a. Exemplar image sequences were generated in Fiji (Schindelin et al., 2012). Quantification of p65 response amplitude used ROI measurement of fluorescent signal in the nucleus and cytoplasm of cells prior to stimulation and at the apex of the first peak. Nuclear and cytoplasmic fluorescence tracking of LMB treatment experiments used CellTracker software (Shen et al., 2006).

### Fluorescence Correlation Spectroscopy (FCS)

Cells were seeded into 35 mm glass-bottomed dishes and imaged using a Zeiss LSM 880 microscope, as described above.

Fluorescence fluctuations were recorded in five separate measurements of 5 s for manually selected discrete locations in either the cytoplasm or nucleus across many cells, with the pinhole set to measure 1 AU, the equivalent of 0.75 fL volume when using 488 nm laser. Data was analysed using the “Fish-and-Cushion” software as described in (Koch et al., 2022). This performs autocorrelation analysis, which determines the concentration of fluorescent protein by selecting parameters from the best fit model across a range of models that capture protein mobility and photochemistry fluctuations for each cell measurement.

### Nanostring

RNA was isolated as described above. Samples were analysed with the Nanostring nCounter analysis system and a custom CodeSet (Supplementary Table S3). Data was processed in nSolver Analysis Software v4.0, with normalisation to five housekeeping genes and internal positive control probes. Clustering analysis grouped genes based on Pearson correlation coefficient of log count values, with linkage calculated from average distance between elements. Nanostring data is available from the GEO repository (accession GSE228526).

### ChIP

Cells were seeded into 150 mm dishes (3M/dish) and allowed to grow until near confluent. Cells were treated with TNF⍺ +/− LMB as described, then processed using the EZ-Magna ChIP kit (Millipore 17–409). Chromatin was fragmented by sonication at 4°C using a Bioruptor 300 (Diagenode; 45 cycles, 30 s on/30 s off). Immunoprecipitation used 4 μg ChIP validated antibody against NF-κB p65 (Millipore, 17–10060), or control antibodies against polII or IgG. Chromatin immunoprecipitation was quantified by qPCR.

### Animals

Mice were maintained in the University of Manchester Biological Services Facility. All protocols were approved by the University of Manchester Animal Welfare and Ethical Review Body and licenced under the Animals (Scientific Procedures) Act 1986.

### Generation of transgenic mice

To create BAC transgenic mice 10 μg maxi-prepped BAC DNA (Nucleobond 100) was linearised by restriction digest (NotI) and purified by sepharose column purification (GE Healthcare). Briefly, a standard 5 mL pipette was used as a column, with the cotton plug removed and filled with injection buffer (sterile filtered 10 mM Tris (pH 7.5), 0.1 mM EDTA (pH 8.0), 100 mM NaCl) equilibrated sepharose beads. Linearised DNA, with bromophenol blue dye, was added to the column, and once the dye had entered the beads more injection buffer added. Fractions were collected every 5 minutes until dye had drained from the column. A sample of each fraction was run on an agarose gel to confirm DNA purity, and the DNA containing fraction was diluted to 2 ng/μL for mouse zygote injection.

Zygote injections, in C57/BL6j background embryos, were performed by the Manchester Genome Editing Unit. Pups were genotyped using primers specific to the fluorescent protein gene. The strain is cryopreserved and available on request.

### Targeted locus amplification genotyping

Mice aged 6–8 weeks were culled by an S1 method. The spleen was removed and prepared as described in the Cergentis spleen sample preparation protocol. Samples were sent to Cergentis (Utrecht, Netherlands) for identification of transgene integration site and copy number analysis.

### Statistical analysis

Statistical analysis used GraphPad Prism. Details of sample size and statistical tests are provided in figure legends.

## Results

### Generation and characterisation of clonal SK-N-AS IκB⍺-eGFP BAC cell lines

**FIGURE 1:**
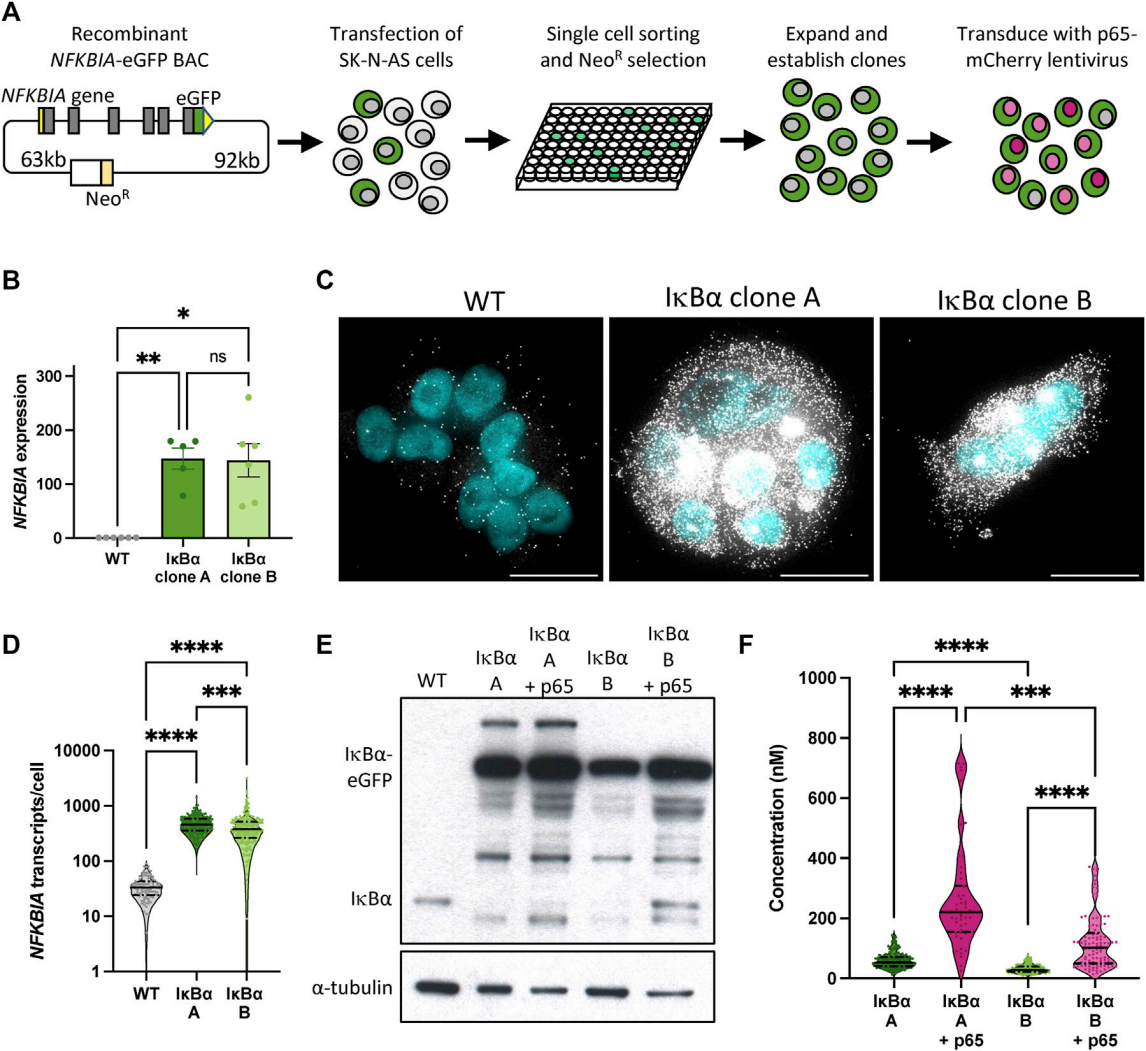
Generation and characterisation of IκB⍺-eGFP reporter cells. **(A)** Schematic representation of cell line generation. Clonal cell lines with integration of a BAC expressing an IκB⍺-eGFP fusion protein were selected. Validated clonal cell lines were subsequently transduced with p65-mCherry lentivirus. **(B)** qRT-PCR measurement of *NFKBIA* gene expression in unstimulated conditions. Expression is normalised to *PPIA*. Error bars indicate mean±SEM. N = 5-6, One-way ANOVA, Kruskal–Wallis test, Dunn’s multiple comparison correction. **(C)** smRNA-FISH detection of *NFKBIA* transcripts in unstimulated conditions. Probe is shown in white; nuclei counterstained with DAPI are shown in cyan. Scale bar = 20 µm. **(D)** Violin plots of *NFKBIA* transcript numbers quantified by smRNA-FISH detection in clonal cell lines in unstimulated conditions. N = 100–200 cells, imaged over 2-3 independent experiments. One-way ANOVA, Kruskal–Wallis test, Dunn’s multiple comparison correction. Quartile range and median are indicated by dashed/solid lines. **(E)** Protein quantification by Western blot in clonal cell lines in unstimulated conditions. **(F)** Violin plots of cytoplasmic protein quantification by FCS in clonal cell lines in unstimulated conditions. N = 50–200 cells, imaged over 3-4 independent experiments. Quartile range and median are indicated by dashed/solid lines. One-way ANOVA, Kruskal–Wallis test, Dunn’s multiple comparison correction. Throughout, * indicates *p* < 0.05, ** indicates *p* < 0.01, *** indicates *p* < 0.001, **** indicates *p* < 0.0001.

We have previously described the generation of a recombinant BAC expressing an IκB⍺-eGFP fusion protein a pseudo-native genomic context (Adamson et al., 2016). This includes over 150 kb of flanking, intronic and UTR sequence to ensure gene expression is subject to natural regulatory processes and feedbacks. We generated a series of clonal SK-N-AS neuroblastoma cell lines by single cell sorting and antibiotic selection (Figure 1A), and identified two clones, here termed IκB⍺ A and IκB⍺ B (previously named clone C9), (Adamson et al., 2016), with elevated IκB⍺ expression levels. In order to visualise p65 in these backgrounds, these clones were also transduced with a lentiviral p65-mCherry expression vector (Figure 1A).

We used a range of approaches to quantify IκB⍺ expression in these clones. Quantitative RT-PCR indicates levels of IκB⍺ transcript far in excess of WT cells (Figure 1B). Using probes directed against IκB⍺ we identified between 10 and 100 IκB⍺ transcripts per cell in unmodified (WT) SK-N-AS cells by single molecule RNA fluorescence *in situ* hybridisation (smFISH; Figure 1C). In comparison, we detected between 100 and 1,000 transcripts per cell in the BAC clones (mean of 480 transcripts per cell in clone A, 434 in clone B; Figure 1D), typically densely clustered around a single bright spot, representing the site of active transcription from the integrated BAC constructs. This may be an underestimation of transcript number since saturated signal detection at the sites of transcription meant transcripts could not be individually resolved at these sites. Western blot analysis of protein extracts revealed elevated IκB⍺-eGFP levels in both clones, with and without p65-mCherry (Figure 1E). We detect a number of additional protein bands which may represent degraded or intermediate products in the overexpression cell lines. We also observed a reduction in the level of endogenous, untagged IκB⍺ protein in both overexpression clones. Finally, we used Fluorescence Correlation Spectroscopy (FCS) to measure the intracellular concentration of IκB⍺-eGFP. In IκB⍺ clone A, FCS determined the mean cytoplasmic concentration of IκB⍺-eGFP to be 59 nM ( ± 26 nM), compared to 32 nM ( ± 13 nM) in IκB⍺ clone B. Cytoplasmic and nuclear protein diffusion rates were comparable between clones (Supplementary Figures S1A, B). In both clones, co-overexpression of p65-mCherry resulted in increased expression of IκB⍺-eGFP (Figure 1F; protein interaction measured in Supplementary Figure S1C), corroborating previous studies (Lee et al., 2014). These approaches all confirm that clones A and B exhibit elevated IκB⍺ levels compared to WT cells, with Clone A showing higher protein expression levels than Clone B.

### IκB⍺ oscillation dynamics are independent from IκB⍺ expression level

IκB⍺ is an early target gene responsive to stimulation by TNF⍺ treatment (Hao and Baltimore, 2009). We found overall levels of IκB⍺ transcript in our BAC clonal cell populations to be elevated even in the absence of TNF⍺ stimulation (Figure 1), so next we used smFISH to characterise the effect of TNF⍺ stimulation on transcript levels in single cells (Supplementary Figure S2A). In untreated WT cells we detected a low background level of cytoplasmic IκB⍺ mRNA, which increased significantly after 130 min TNF⍺ treatment (Supplementary Figure S2A, upper panels, and Supplementary Figure S2B). One or two distinct bright signal spots, indicating the allelic sites of transcription, are seen in the nuclei of treated cells (Supplementary Figure S2A, upper panels). Treatment of cell lines with TNF⍺ resulted in detection of extremely high transcript levels, suggesting further induction of expression over the high basal level in untreated clonal cells (and prohibiting accurate quantitative analysis of transcript numbers). Co-staining for *eGFP* transcript sequences confirms that most cellular transcripts are derived from the integrated BAC *NFKBIA-eGFP* transgene. This indicates the integration of IκB⍺-eGFP BACs results in significant overexpression of IκB⍺ transcript when compared to WT cells, in both untreated and treated conditions.

**FIGURE 2:**
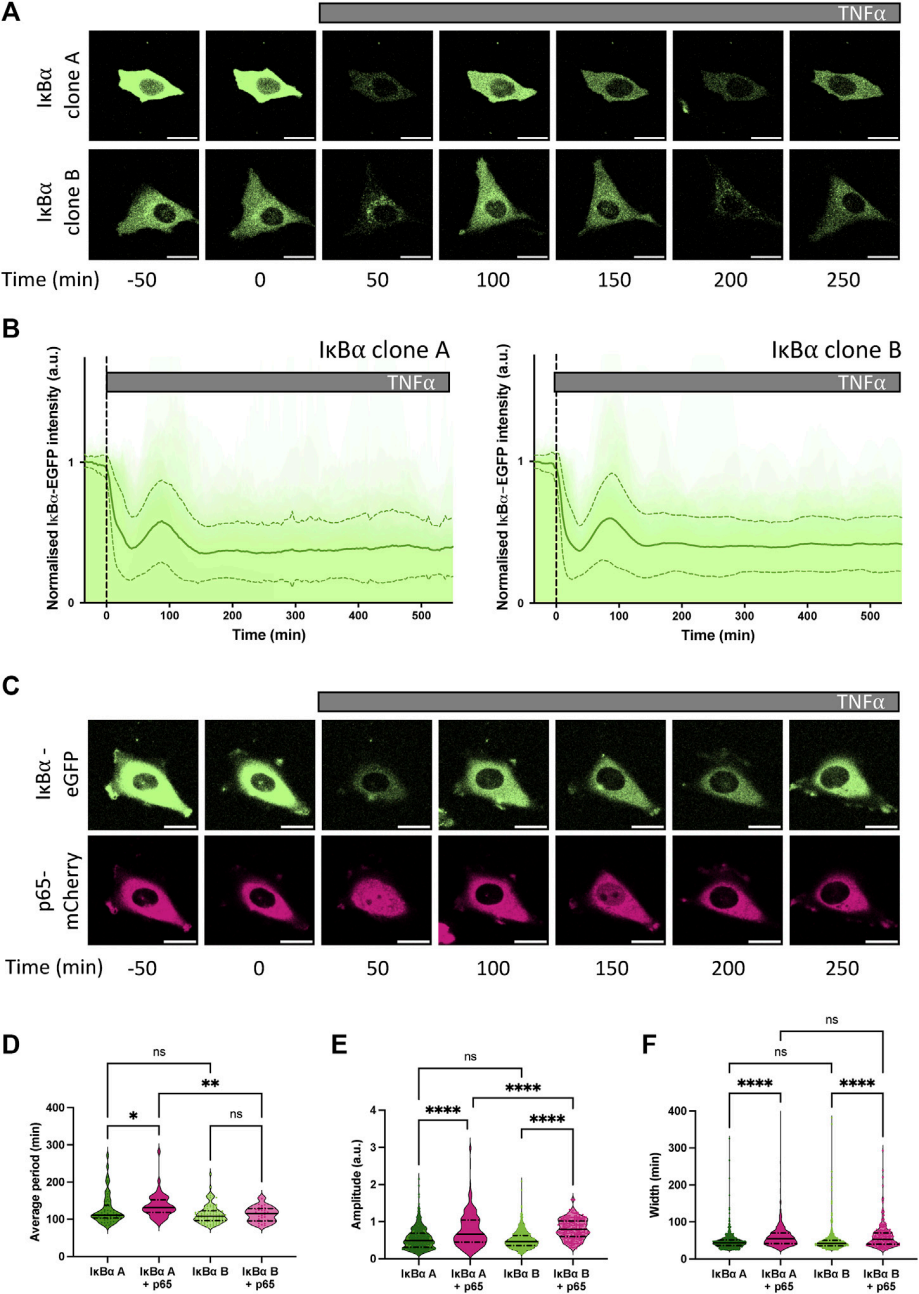
Dynamic response of IκB⍺-eGFP reporter cells to TNF⍺ treatment. **(A)** Example time course images of IκB⍺-eGFP clonal cell response to TNF⍺ treatment. Scale bar = 20 µm. **(B)** Clonal cell response to continuous TNF⍺ treatment. Individual traces are shown in pale green; population average ± SD is shown in dark green. N = 140–160 cells imaged over at least 6 independent experiments. **(C)** Example time course images of IκB⍺-eGFP clonal cells transduced with p65-mCherry lentivirus response to TNF⍺ treatment. Scale bar = 20 µm. **(D–F)** Quantification of dynamic cell response to continuous TNF⍺ treatment. Normalised fluorescence intensity was tracked over at least three oscillation cycles and traces quantified to determine average period of oscillation **(D)**, peak amplitude **(E)** and peak width **(F)**. N = 30–90 cells/clone imaged over at least 2 independent experiments. Quartile range and median are indicated by dashed/solid lines. One-way ANOVA, Kruskal–Wallis test, Dunn’s multiple comparison correction.

Our previous studies have found p65 oscillates from cytoplasm to nucleus in the continuous presence of pro-inflammatory stimuli such as TNF⍺ (Nelson et al., 2004; Ashall et al., 2009). We have also shown, through mathematical modelling and single cell imaging of cells transfected with the IκB⍺-eGFP BAC, that IκB⍺ degrades and resynthesizes out-of-phase to p65 nuclear movements, as expected in a negative feedback regulatory loop (Nelson et al., 2004; Adamson et al., 2016). IκB⍺ oscillations have been observed in several cell types tested, including primary cells derived from transgenic mice (Harper et al., 2018). We next examined the dynamic inflammatory response of our stable cell lines to TNF⍺ by live cell confocal microscopy. At the protein level, continuous TNF⍺ treatment resulted in rapid degradation of IκB⍺-eGFP in both clones (median trough around 30 min), followed by resynthesis of IκB⍺-eGFP with an initial peak 90–110 min after treatment (Figures 2A, B). Cells continued to show oscillations in fluorescence intensity as a result of IκB⍺-eGFP degradation and synthesis, quickly becoming asynchronous (Supplementary Video S1). Response to a short pulse of TNF⍺ demonstrates that IκB⍺ degradation and resynthesis rates are consistent between cell lines, and comparable to unmodified cells (Supplementary Figures S3A, B). Analysis of oscillatory behaviour in clones transduced with p65-mCherry confirmed p65 nuclear translocation occurs out-of-phase with IκB⍺ degradation/resynthesis cycles (Figure 2C), as seen previously (Adamson et al., 2016). Median oscillatory period was 111 min in IκB⍺ clone A and 108 min in IκB⍺ clone B (Figure 2D). This corroborates data previously generated using other NF-κB expression systems, including overexpression of p65 fusions from constitutively active promoters in exogenous vectors (Adamson et al., 2016; DeFelice et al., 2019; Son et al., 2022), from endogenously tagged alleles in MEFs derived from a GFP-p65 transgenic mouse (Zambrano et al., 2016), and from a CRISPR targeted allele in MCF7 cells (Stewart-Ornstein and Lahav, 2016). Analysis of clones transduced with p65-mCherry found a slightly increased median period in clone A cells (131 min), although median period of transduced clone B cells (115 min) was not significantly lengthened compared to untransduced cells (Figure 2D). Amplitude of IκB⍺-eGFP oscillation and peak width were increased by p65-mCherry expression in both clones (Figures 2E, F), but p65-mCherry translocation amplitude was only weakly related to protein expression level (Supplementary Figures S3C, D). This is consistent with previous work, which found that cells maintain a robust oscillatory period of 100–110 min when IκB⍺ or p65 are overexpressed in isolation, but that overexpression of p65 and feedback-responsive IκB⍺ can result in lengthening of the oscillatory period (Nelson et al., 2004). Previous experiments with IκB⍺ under a non-native 5xNF-κB response element promoter resulted in lengthening of the period to around 200 min; however, this system is unlikely to reflect all regulatory inputs as effectively as the pseudo-native context provided by our BAC reporter system.

### IκB⍺ overexpression modulates NF-κB target gene transcription

**FIGURE 3:**
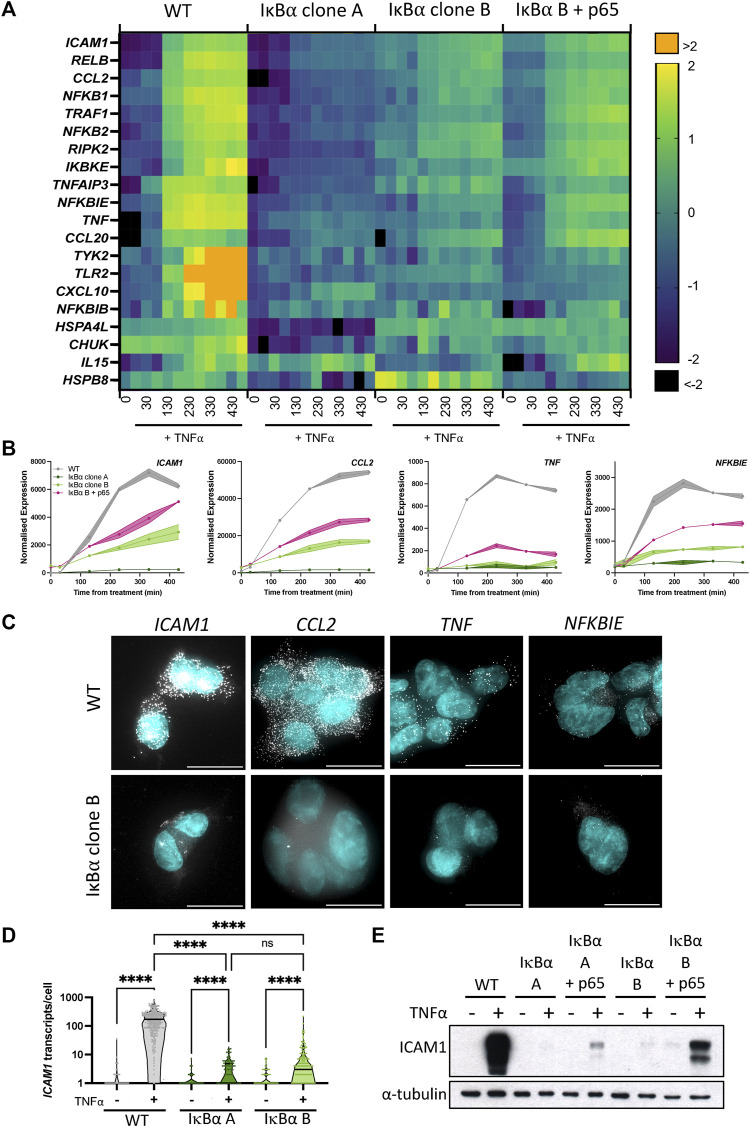
Overexpression of IκB⍺ suppresses target gene expression. **(A)** Heatmap representation of gene expression of clonal cell lines in response to TNF⍺ treatment, as determined by Nanostring gene expression assay. N = 2/time/cell line. Expression is normalised to five housekeeping genes and internal control probes, then scaled to average gene expression level. **(B)** Normalised Nanostring counts for selected genes from **(A)**, mean±error. **(C)** Example images showing smRNA-FISH detection of target gene transcripts in cell lines after 130 min TNF⍺ treatment. Scale bar = 20 µm. **(D)** Quantification of *ICAM1* transcripts by smRNA-FISH in cell lines before and after treatment with TNF⍺ for 130 min. N = 90–300 cells/condition, imaged over six independent experiments. Two-way ANOVA, Tukey’s multiple comparison correction. **(E)** Quantification of ICAM1 protein expression before and after treatment with TNF⍺ for 230 min.

We and others have shown an association between oscillatory behaviour of the NF-κB signalling system and downstream gene expression (Ashall et al., 2009; Martin et al., 2020). Given overexpression of IκB⍺ did not perturb oscillatory behaviour, we investigated whether expression of NF-κB target genes was affected. Wild type (WT) SK-N-AS cells, IκB⍺ Clone A, IκB⍺ Clone B and IκB⍺ Clone B + p65-mCherry transduced cells were treated with TNF⍺ and RNA extracted over a time course. Transcript abundance for a pre-defined NF-κB target gene subset was quantified by Nanostring analysis (Supplementary Table S1). Hierarchical cluster analysis broadly grouped target gene response into four patterns of behaviour (Supplementary Figure S4). The largest cluster, Cluster 1, includes prototypical inflammatory mediators and NF-κB feedback genes which show sustained activation over 430 min in unmodified SK-N-AS cells (Figure 3A). In IκB⍺ clone A cells, TNF⍺ treatment resulted in substantially reduced activation of these gene targets. IκB⍺ clone B cells showed a strong dampening of activation, which could be partially rescued by co-overexpression of p65-mCherry in lentivirus transduced cells. Genes in Cluster 3 showed basal upregulation in response to IκB⍺ overexpression in the clonal cell lines, whilst genes in other clusters demonstrated inter-clone variability. We focused on genes from Cluster 1 for further investigation.

We validated the Nanostring results using complementary approaches for several genes showing behaviour typical of Cluster 1: *ICAM1, CCL2, TNF* and *NFKBIE* (Figure 3B). These genes have been characterised as direct targets of NF-κB signalling and are upregulated in response to inflammatory cytokine treatment (Sutcliffe et al., 2009; Ghosh et al., 2010; Astarci et al., 2012). Transcript level of target genes was quantified by smFISH, following treatment with TNF⍺ for 130 min (Figure 3C; treated cells for WT and IκB⍺ clone B shown). Consistent with the gene expression data from Nanostring analysis, WT cells exhibited an increase in transcript number after treatment, but IκB⍺ clone B cells showed comparatively low transcript levels for all gene targets. In common with previous studies, numbers of transcripts per cell showed considerable heterogeneity for some targets, e.g., *TNF* (Bagnall et al., 2018; Bagnall et al., 2020; Bass et al., 2021)*.* Quantification of transcript number found WT cells express 176–210 *ICAM1* transcripts per cell after 130 min TNF⍺ stimulation (95% confidence interval, CI), whilst IκB⍺ clone A expressed an average of 3 (95% CI 2–4) transcripts per cell, and clone B expressed an average of 10 (95% CI 6–13) transcripts per cell (Figure 3D). Western blot analysis confirmed strong protein expression of ICAM1 in TNF⍺-treated WT cells, but undetectable and near-undetectable levels of ICAM1 protein in clones A and B respectively (Figure 3E). Again, the expression response could be partially rescued by co-overexpression of p65-mCherry (Figure 3E). These data indicate that whilst IκB⍺ protein oscillations and p65 N:C translocations are robust to changes in IκB⍺ level, overexpression of IκB⍺ can result in profound effects on target gene activation. NF-κB activity is regulated by multiple feedback loops, and previous work has hypothesised IκB⍺ feedback dictates the amplitude of response, with other feedbacks (e.g., A20) altering timing (Fagerlund et al., 2015; Adamson et al., 2016). We find that, in our system, response timing is unaltered but the ‘amplitude’ of response (i.e., activation of target genes) has been dampened by IκB⍺ overexpression.

### IκB⍺-eGFP overexpression leads to elevated nuclear IκB⍺ and competition with target DNA response elements for nuclear p65

The correlation between elevated IκB⍺ expression and canonical target gene repression, together with partial gene expression rescue by increased p65 concentration, indicates these effects are likely to be a response to variation in intracellular protein concentration. Mechanistic modelling has previously found that sensitivity to TNF⍺ is dependent upon the cellular ratio of NF-κB to its inhibitor, and nucleocytoplasmic shuttling of these complexes in resting cells (Patel et al., 2021). IκB⍺ has both nuclear import and export sequences, and the equilibrium between these two processes determines its net distribution.

**FIGURE 4:**
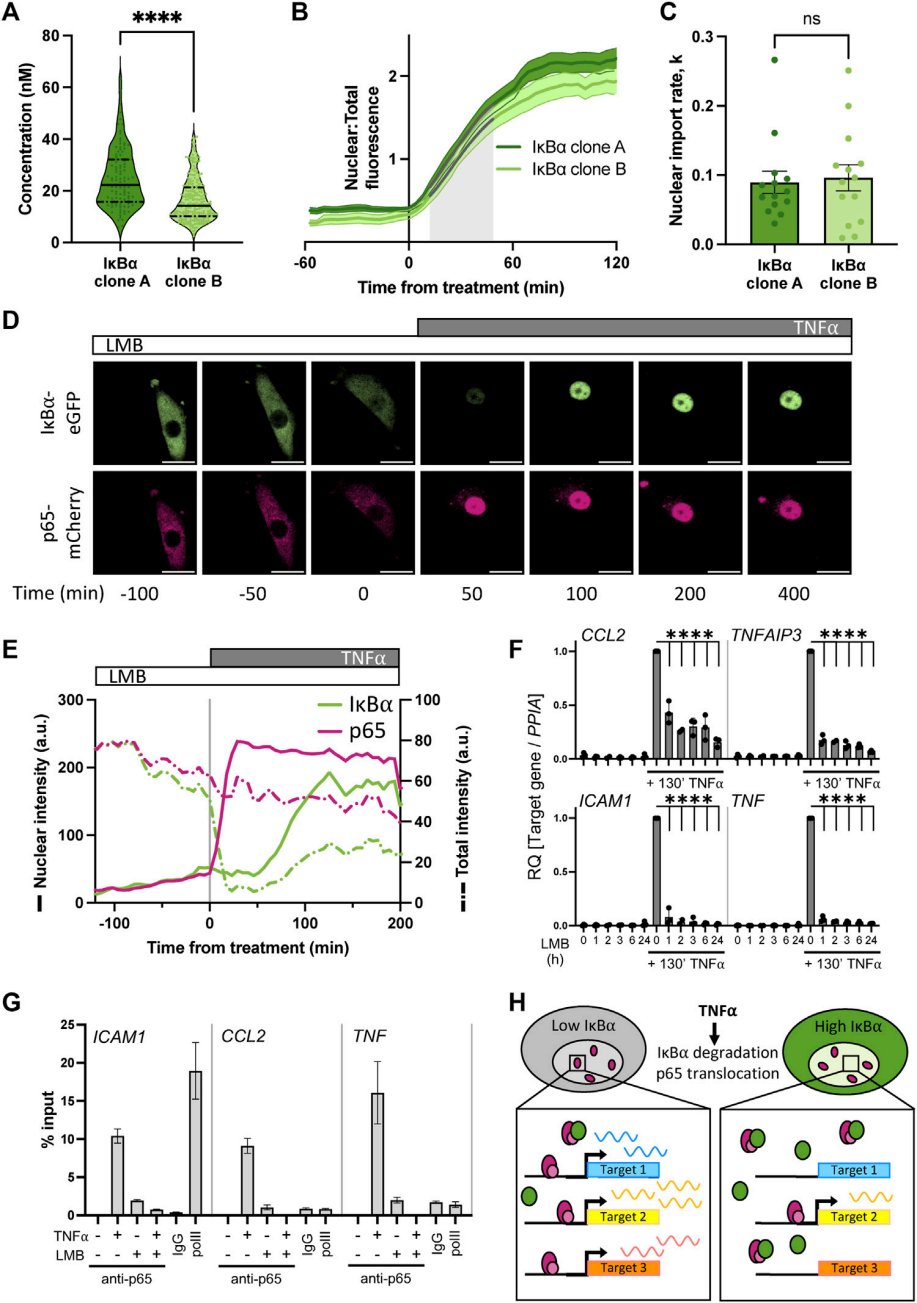
Nuclear accumulation of IκB⍺ suppresses target gene expression. **(A)** FCS quantification of nuclear protein in clonal cell lines in unstimulated conditions. Quartile range and median are indicated by dashed/solid lines. Mann-Whitney test, N = 100–250 cells over 3 independent experimental replicates. **(B)** Quantification of nuclear fluorescence (mean±SD) following LMB treatment of cells. Import rate was calculated from 10 to 50 min after treatment (shaded grey) from N = 14 cells/clone. **(C)** IκB⍺ nuclear import rate following LMB treatment, calculated from nuclear fluorescence data in **(B)** using a non-linear logistic growth model with least squares fit (indicated by grey lines on B). Mann-Whitney test. **(D)** Example time course images of cell response to LMB treatment (120 min) followed by subsequent addition of TNF⍺. Scale bar = 20 µm. **(E)** Example cell fluorescence tracking showing quantification of response to LMB followed by subsequent addition of TNF⍺. Nuclear intensity is indicated by unbroken lines, total cell intensity is indicated by dashed lines. **(F)** qRT-PCR measurement of SK-N-AS target gene expression response to TNF⍺ treatment following LMB preincubation for 1–24 h. Target gene expression was normalised to *PPIA*. N = 3/treatment/time, mean±SD. Two-way ANOVA, Dunnett’s multiple comparison test. **(G)** Chromatin immunoprecipitation using anti-p65 antibody against binding sites in the promoter regions of indicated target genes, with or without LMB pretreatment (120 min) or TNF⍺ treatment (130 min). Samples for IgG and polII immunoprecipitations were treated with TNF⍺ for 130 min. N = 2/treatment, mean ± error. **(H)** Schematic representation of model of IκB⍺ action on target gene expression.

We and others have previously shown that IκB⍺ accumulates in the nucleus upon cellular treatment with the nuclear export inhibitor Leptomycin B (LMB) (Rodriguez et al., 1999; Nelson et al., 2004; Sung et al., 2009), indicating that subcellular localisation of IκB⍺ is dynamic. Such an equilibrium has previously been found for NF-κB:IκB⍺ complexes (Huang et al., 2000); we hypothesise that under basal conditions a similar equilibrium exists between IκB⍺ nuclear export and import in our cells. This results in an elevated basal concentration of nuclear IκB⍺ in our overexpressing cells. To confirm this, we used FCS to measure the concentration of fluorescent IκB⍺-eGFP in the nucleus of the BAC clonal cell lines (Figure 4A). We detected a nuclear presence of IκB⍺ in both clones, with a slightly higher concentration for IκB⍺ clone A (25 nM vs. 16 nM). Treating cells with LMB to block nuclear export resulted in accumulation of IκB⍺-eGFP in the nucleus of both clones (Figure 4B). The rate of import was similar in each clone (Figure 4C), indicating this rate is unaffected by IκB⍺ expression level. These data suggest the proportion of IκB⍺ molecules in the cytoplasm vs. nucleus of a given cell is correlated (as previously observed in (Kardynska et al., 2018)), and nuclear import/export maintains this equilibrium, resulting in nuclear concentration of IκB⍺ being a fixed proportion of total cellular IκB⍺. As clone A cells have higher overall IκB⍺ protein expression levels (Figure 1), this results in higher nuclear IκB⍺ levels in comparison to clone B (Figure 4A). Thus, in our system, the BAC-mediated increase in IκB⍺ expression results in an increase in nuclear IκB⍺ concentration.

Excess nuclear IκB⍺ could potentially act as a competitor molecule to p65 action on target gene activation after TNF⍺ treatment by preventing p65 binding to target gene promoters. We therefore investigated the effect of deliberately increasing IκB⍺ nuclear concentration by LMB treatment prior to TNF⍺ treatment. IκB⍺ clone B cells transduced with p65-mCherry were treated for 2 hours with LMB and imaged by time lapse confocal microscopy (Figure 4D). Both the labelled IκB⍺ and p65 proteins were observed to accumulate in the nucleus (Figures 4D, E; solid green line). Upon stimulation with TNF⍺, the remaining cytoplasmic IκB⍺ was rapidly degraded, resulting in release of p65 to translocate to the nucleus. Notably, IκB⍺-eGFP which had already translocated to the nucleus in the presence of LMB wasn’t degraded by stimulation with TNF⍺. We observe slight photobleaching over the experiment, but this typically represents only 5%–10% total fluorescence and does not obscure the dynamics of IκB⍺ localisation over the timescales we consider (Supplementary Figure S5). This confirms that nuclear IκB⍺ is protected from cytoplasmic signalling (Rodriguez et al., 1999). A transcriptional cycle was activated, evidenced by rise in total IκB⍺-eGFP levels, and newly synthesised IκB⍺-eGFP rapidly localises to the nucleus, but does not export p65 to the cytoplasm due to the continued presence of LMB (Figure 4E). Despite the continued presence of p65 in the nucleus no further IκB⍺-eGFP is detectably produced, indicating that p65 is now transcriptionally inactive. This may be due to IκB⍺ complexing with p65 and removing it from the IκB⍺ BAC transgene (and other target genes), although we cannot discount a role for dephosphorylation and inactivation of p65 through post-translational modification.

We hypothesised that increasing the concentration of IκB⍺ could result in dampened downstream p65 target gene activation due to either 1) competitive binding by elevated nuclear IκB⍺ to nuclear translocated p65, preventing binding and activation of target genes, or 2) elevated cytoplasmic IκB⍺ resulting in incomplete IκB⍺ degradation in response to TNF⍺ treatment, reducing the level of free p65 available for nuclear translocation. To test whether elevating nuclear IκB⍺ levels can repress p65 target gene activation, we treated WT SK-N-AS cells with LMB to perturb the nuclear:cytoplasmic IκB⍺ equilibrium and result in accumulation of IκB⍺ and p65 in the nucleus. Following a range of periods of LMB treatment, we stimulated cells with TNF⍺ for 130 min and examined the impact on downstream gene expression. After LMB pre-incubation and TNF⍺ stimulation, mRNA was extracted and target gene expression was analysed by qRT-PCR. LMB treatment for up to 24 h did not result in target gene activation despite the nuclear accumulation of p65 (Figure 4F), which corroborates previous findings (Sung et al., 2009). TNF⍺ treatment in the absence of LMB resulted in strong target gene activation; this was significantly abrogated in cells subjected to pre-incubation with LMB, resulting in a marked reduction in target gene expression for all analysed targets after as little as 1 hour of LMB pre-treatment.

It is known that p65 activates target genes by binding NF-κB response elements (REs) in promoters, which facilitates the recruitment of transcriptional machinery (Zhang et al., 2017). If nuclear IκB⍺ competitively prevents p65 binding to target sites in the genome, we would expect to see reduced p65 occupancy on NF-κB REs. Previous work in mouse B cells has found that increased nuclear occupancy of IκB⍺, in this study a result of nuclear export sequence (NES) mutation, reduced NF-κB recruitment to κB-containing probes on electrophoretic mobility shift assay (Wuerzberger-Davis et al., 2011). We used chromatin immunoprecipitation (ChIP) to investigate recruitment of p65 to well-characterised NF-κB REs in target gene promoters in WT cells. TNF⍺ treatment for 130 min resulted in strong recruitment of p65 to all target gene promoters in the absence of LMB (Figure 4G). Interestingly, we also detected a low level of recruitment of p65 when cells were treated with LMB alone, indicating that nuclear p65 retains some capacity to bind target promoters whilst transcriptionally inactive. Following pre-treatment with LMB, 130 min of TNF⍺ treatment did not result in p65 binding to target promoters (Figure 4G). This indicates that disrupting the equilibrium between nuclear and cytoplasmic localisation has a potent effect on gene activation. Overall, this supports our hypothesis that increasing the nuclear concentration of IκB⍺ results in dampened downstream p65 target gene activation due to competitive binding by nuclear IκB⍺ to translocated p65, reducing target gene activation (Figure 4H).

### An *in vivo* BAC IκB⍺ overexpression model confirms the potency of nuclear IκB⍺ to repress inflammatory gene activation

**FIGURE 5:**
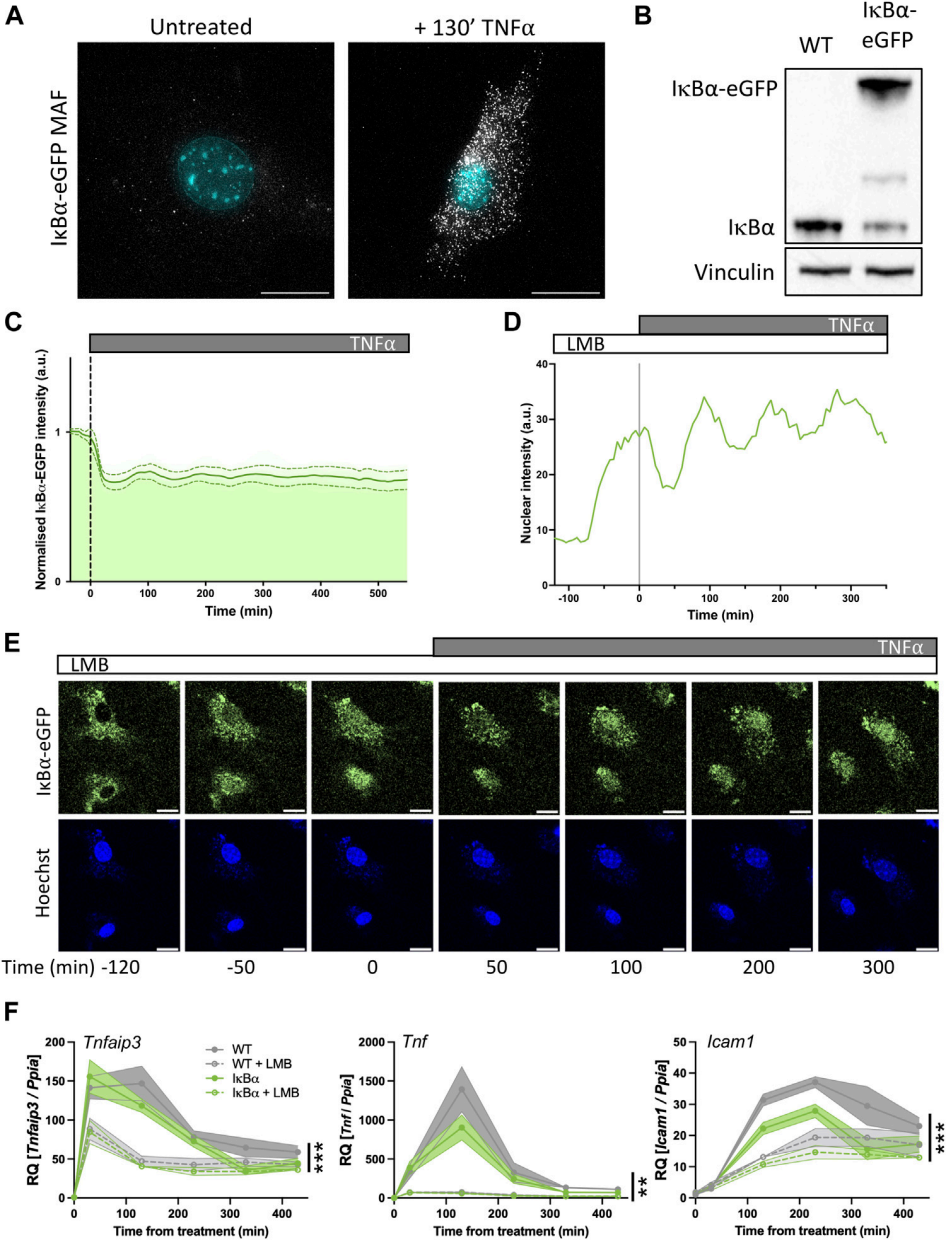
Primary mouse cell target gene expression is sensitive to nuclear IκB⍺ accumulation. **(A)** smRNA-FISH quantification of RNA transcripts in mouse adult fibroblast (MAF) cells. **(B)** Western blot quantification of IκB⍺ protein in MAF cells. **(C)** Quantification of live cell nuclear fluorescence in IκB⍺-eGFP MAF cells in response to TNF⍺ treatment. N = 16 cells. **(D)** Example cell fluorescence tracking of live cell nuclear fluorescence in IκB⍺-eGFP MAF cells in response to LMB treatment. **(E)** Example time course images of MAF response to LMB treatment (120 min) followed by subsequent addition of TNF⍺. Cell nuclei were stained with Hoechst. Scale bar = 20 µm. **(F)** qRT-PCR measurement of MAF gene expression response to TNF⍺ treatment following LMB pretreatment for 120 min. Target gene expression was normalised to *Ppia*. N = 3/treatment/time/genotype, represented as mean±SEM. Three-way ANOVA with Geisser-Greenhouse correction; effect of LMB treatment is indicated to right of curves.

We used the IκB⍺-eGFP BAC to generate a transgenic mouse line to test this *in vivo*. Our transgenic line contains the IκB⍺-eGFP BACs at a single integration site on chromosome 8, with an estimated integration copy number of 2–6 (Supplementary Figure S6). In primary fibroblasts from these mice, smFISH analysis indicates low IκB⍺ transcript levels in the absence of treatment, with an induction after treatment with TNF⍺ (Figure 5A). Transcript expression level appears lower than observed in our BAC clonal cell lines, and more comparable to WT SK-N-AS cells, likely as a result of the low copy number integration.

Western blot analysis showed a detectable band at native IκB⍺ size in both WT mice and IκB⍺-eGFP BAC mice. Expression level of untagged endogenous IκB⍺ appeared reduced compared to WT mice (around 30% of WT level, contrasting with the almost complete loss seen in the clonal cell lines), and we detect a strong higher molecular weight IκB⍺-eGFP band in the transgenic mice, resulting in total IκB⍺ levels being around 1.8 times higher overall (Figure 5B). Upon TNF⍺ treatment of the IκB⍺-eGFP BAC fibroblasts we detect oscillation cycles of degradation and resynthesis (with an initial peak at around 100 min after treatment; Figure 5C). We tested the effect of LMB treatment upon these cells and found, as with the clonal cell lines, that LMB treatment resulted in nuclear accumulation of the IκB⍺-eGFP fusion protein within 2 h (Figures 5D, E). We see some residual oscillation in IκB⍺-eGFP nuclear intensity, which may indicate that inhibition of p65 activity as a result of nuclear IκB⍺ accumulation is not complete in these cells, possibly due to lower levels of IκB⍺ protein expression. This would result in reduced transcription of target genes, but not complete suppression.

To test this, we investigated the impact of LMB treatment and/or IκB⍺ overexpression on the target genes *Tnfaip3, Tnf* and *Icam1* by RT-qPCR. IκB⍺ overexpression resulted in reduced activation of *Tnf* and *Icam1*, but not *Tnfaip3,* after TNF⍺ treatment. When cells were pre-treated with LMB, fibroblasts derived from both WT and IκB⍺-eGFP BAC mouse models exhibited lower activation of all three targets, with a pronounced loss of *Tnf* gene activation. These data corroborate the findings in BAC cell lines, showing that nuclear IκB⍺ can have a suppressive effect upon NF-κB activation of target genes.

## Discussion

We have used a stably-integrated BAC reporter construct to investigate the dynamic response of the canonical NF-κB signalling proteins to cytokine stimulation in human and mouse cells. We find that, across a range of expression levels, p65 and IκB⍺ reporter proteins show robust oscillatory dynamics. However, although we find protein dynamics in these systems to be insensitive to reporter overexpression, we find the transcriptional response of well-characterised NF-κB target genes to be broadly suppressed. This suppression is IκB⍺ dose-dependent, and can be partially mitigated by overexpression of p65. We hypothesise that target gene transcriptional reduction results from increased levels of nuclear IκB⍺, and show that artificial increase of nuclear IκB⍺ level, by treatment with the nuclear export inhibitor LMB, similarly suppresses target gene expression.

The NF-κB-IκB⍺ signalling interaction is considered a canonical example of negative feedback regulation (Prescott et al., 2021). In our reporter model, we see high levels of IκB⍺ transcript in basal and treated conditions (Figures 1B–D; Supplementary Figure S2). As IκB⍺ is a prominent negative regulator in the NF-κB signalling system, high transcript availability might be expected to result in rapid production of new IκB⍺ protein, shortening the interval between cytokine-induced NF-κB activation and subsequent repression through retrieval of p65 from the nucleus to the cytoplasm. Mathematical models from our group and others predict this would be the case; however, here we observed limited effects on NF-κB dynamics, such that cells with elevated IκB⍺ maintained a near 100 min oscillatory period. This may indicate that control of oscillatory dynamics can be partially decoupled from feedback gene transcription, and the “inhibitory” arm of the oscillation cycle (i.e., IκB⍺ re-synthesis) is also under post-transcriptional control. As an example, our previous work found the refractory period between activating cytokine treatment and induction of IκB⍺-mediated repression to be determined by a predicted enzymatic activity upstream of IKK activation (Adamson et al., 2016).

One can hypothesise that despite high levels of IκB⍺ transcript, no new protein can accumulate until deactivation of IKK occurs, and thus control of IKK activity may modulate oscillatory period (Fagerlund et al., 2015). In line with this, we have previously shown that manipulation of the level of *TNFAIP3*/A20 alters oscillatory period (Harper et al., 2018) and the refractory period between pulses of stimulation (Adamson et al., 2016). Other computational and experimental analyses have identified nuclear import and export of IκB⍺ as key determinants of the dynamics of post-induction repression (Fagerlund et al., 2015). Our measurements find nuclear import rate to be comparable for both reporter cell lines, irrespective of protein expression level (Figures 4B, C), meaning this parameter does not alter oscillatory period in our reporter cells. More explicit modelling of the effect of nuclear import/export rates on oscillatory dynamics found persistent NF-κB translocation even in the presence of high levels of IκB⍺ (Korwek et al., 2016). Taken together, these studies suggest IκB⍺ level alone is not sufficient to control period length and additional actions, such as IKK activity/signalling and other regulatory feedbacks, combine to regulate cell periodicity.

IκB⍺-deficient mice typically die shortly after birth, displaying signs of skin inflammation and increased expression of inflammatory chemokines (Rebholz et al., 2007). This suggests one key role of IκB⍺ is in preventing “excessive” transcription of inflammatory target genes. We hypothesise that the clear suppressive effect of IκB⍺ overexpression on NF-κB target gene transcription (Figure 3) can be attributed to the increased levels of nuclear IκB⍺ present (Figure 4A). Accumulation of labelled and endogenous IκB⍺ in the nucleus in the absence of cytokine treatment has previously been taken as evidence for a basal level of shuttling between the cytoplasm and nucleus (Johnson et al., 1999; Carlotti et al., 2000), meaning high overall IκB⍺ level is likely to result in higher nuclear levels of IκB⍺. The elevated nuclear IκB⍺ level acts as a potent competitor for translocating active p65, preventing or attenuating promoter binding and effective target gene activation. Biophysical studies have shown that formation of a ternary complex with IκB⍺ decreases the affinity of NF-κB for DNA and promotes formation of the stable, high affinity NF-κB-IκB⍺ complex, resulting in thermodynamically favourable “molecular stripping” of NF-κB from target promoters (Alverdi et al., 2014; Potoyan et al., 2016). Mutation of the PEST interaction residues of IκB⍺ slows removal of NF-κB complexes from DNA and relocation of NF-κB from the nucleus (Dembinski et al., 2017). Regulation of DNA binding/unbinding dynamics has been found to influence transcriptional response coherence (Wang et al., 2018). These mechanisms of competition and molecular stripping may be broadly relevant in transcriptional regulation by other factors.

Mechanisms which promote the nuclear localisation of IκB⍺ have been found to have a repressive effect on NF-κB-regulated targets in a number of previous studies. Pre-treatment of cells with LMB protects IκB⍺ from signal-responsive degradation (Rodriguez et al., 1999). This results in a dose-dependent reduction in binding of NF-κB to κB probe sites in electrophoretic mobility shift assays (EMSAs), and reduces activity in transcriptional reporter assays (Huang et al., 2000). We see a similar effect upon the transcription of endogenous target genes in this study (Figures 4F, 5F), also previously seen elsewhere (Sung et al., 2009). We used chromatin immunoprecipitation to confirm that LMB pre-treatment reduces p65 binding at endogenous target promoters (Figure 4G). Promoting IκB⍺ nuclear localisation by other strategies has been found to have a similar repressive effect upon κB reporter transcription. Overexpression of the receptor kinase GRK5 promotes nuclear accumulation of IκB⍺ as a result of physical interaction and translocation, causing reduced κB-luciferase activity and NF-κB binding to κB probes in response to TNF⍺ treatment (Sorriento et al., 2008). Mutation of the IκB⍺ N-terminal NES also results in nuclear accumulation of IκB⍺ (Wuerzberger-Davis et al., 2011), and these NES mutant mice show reduced expression of some NF-κB subunits and other target genes in response to LPS stimulation, as well as defective B cell maturation and lymph node development, and alterations in T cell development.

We cannot rule out that IκB⍺ may have other effects in this overexpression system, as non-NF-κB-dependent functions have previously been identified. A SUMOylated form of IκB⍺ has been identified in the nucleus of keratinocytes, where it physically interacts with H2A, H4 and PRC2 components including SUZ12 (Mulero et al., 2013). In this context IκB⍺ has a repressive effect on PRC2 target genes, including members of the *HOX* family; IκB⍺ binding at these loci is released by TNF⍺ treatment, resulting in swift upregulation. SUMOylated IκB⍺ has also been detected in the intestinal crypt cells of adult mice, with binding detected at promoters by ChIP-seq (Marruecos et al., 2020). IκB⍺ again associated with PRC2 components in these cells, and knock-out of IκB⍺ resulted in impaired maturation to adult cell identity, improving regenerative properties in response to inflammatory challenge. The effects we observe on the canonical NF-κB target genes of Cluster 1 (Figure 3) is responsive to “rebalancing” via increasing p65 levels; however, effects on other genes might reflect pleiotropic regulation by IκB⍺. Another limitation of our work is that we have not characterised the effect of perturbation of other inhibitors and feedback genes (*NFKBIB, NFKBIE, TNFAIP3*, etc.) on NF-κB-dependent transcription. *NFKBIE* has been shown to influence signalling response heterogeneity, whilst *TNFAIP3* can modulate NF-κB response to physiological conditions (Paszek et al., 2010; Harper et al., 2018). *NFKBIB* overexpression in carcinoma cell lines results in reduced NF-κB binding to DNA and downregulation of target gene transcription, with tumour-suppressive effects (Phoon et al., 2016). The IκB orthologues have non-redundant functions (Clark et al., 2011); further investigation may help to segregate orthologue functionality, but is beyond the scope of this paper. IκB⍺ regulation also occurs post-translationally, and therapeutic intervention with proteasome inhibitors has been considered for diseases involving prominent NF-κB dysregulation (Vrabel et al., 2019; Wang et al., 2020). Introduction of a super-repressor form of IκB⍺ that is resistant to proteolytic degradation improves survival in septic shock mouse models (Choi et al., 2020), suggesting increased IκB⍺ level can have potentially beneficial anti-inflammatory effects in some contexts.

Overexpression of a gene is a classic experimental approach to inform gene and protein function. There are a number of ways in which this can be achieved, including small exogenously delivered constructs (plasmids, lentivirus), or, more recently, via activation of the endogenous gene locus by CRISPR activation (Adli, 2018). However, these methods fail to recapitulate the endogenous regulatory context of the overexpressed gene, so may not accurately reproduce native dynamic properties and responses to extrinsic stimulation. Overexpressing target genes using BACs provides copy number perturbation and increased levels of protein, but maintains regulation of the transgene in a pseudo-genomic context. In this study we used cell line tools and an *in vivo* mouse model that overexpress a labelled IκB⍺ gene from a BAC that responds normally to an inflammatory stimulus. Recent studies have highlighted differences between cells derived from primary tissues with respect to NF-κB signalling dynamics (Rahman et al., 2022), and our mouse would act as a useful physiological tool for investigation of this phenomenon. We used our models to demonstrate that IκB⍺ overexpression has a pronounced effect on inflammatory gene activation, mediated through elevated nuclear IκB⍺ that competes for translocating p65. This results in a broad anti-inflammatory effect without compromising the dynamic NF-κB signalling response to an activating inflammatory cytokine.

## Data Availability

The datasets presented in this study can be found in online repositories. The names of the repository/repositories and accession number(s) can be found below: https://www.ncbi.nlm.nih.gov/geo/, GSE228526.

## Supplementary material

SUPPLEMENTARY VIDEO S1 Time lapse confocal microscopy images of Clone A + p65 cells.
