# Supplementary material for: Overexpression of IκB⍺ modulates NF-κB activation of inflammatory target gene expression
Source: Front Mol Biosci. 2023 May 9;10:1187187. doi: 10.3389/fmolb.2023.1187187 (PMC10203502; doi:10.3389/fmolb.2023.1187187)
Supplement: Supplementary file 1 [file DataSheet1.PDF]

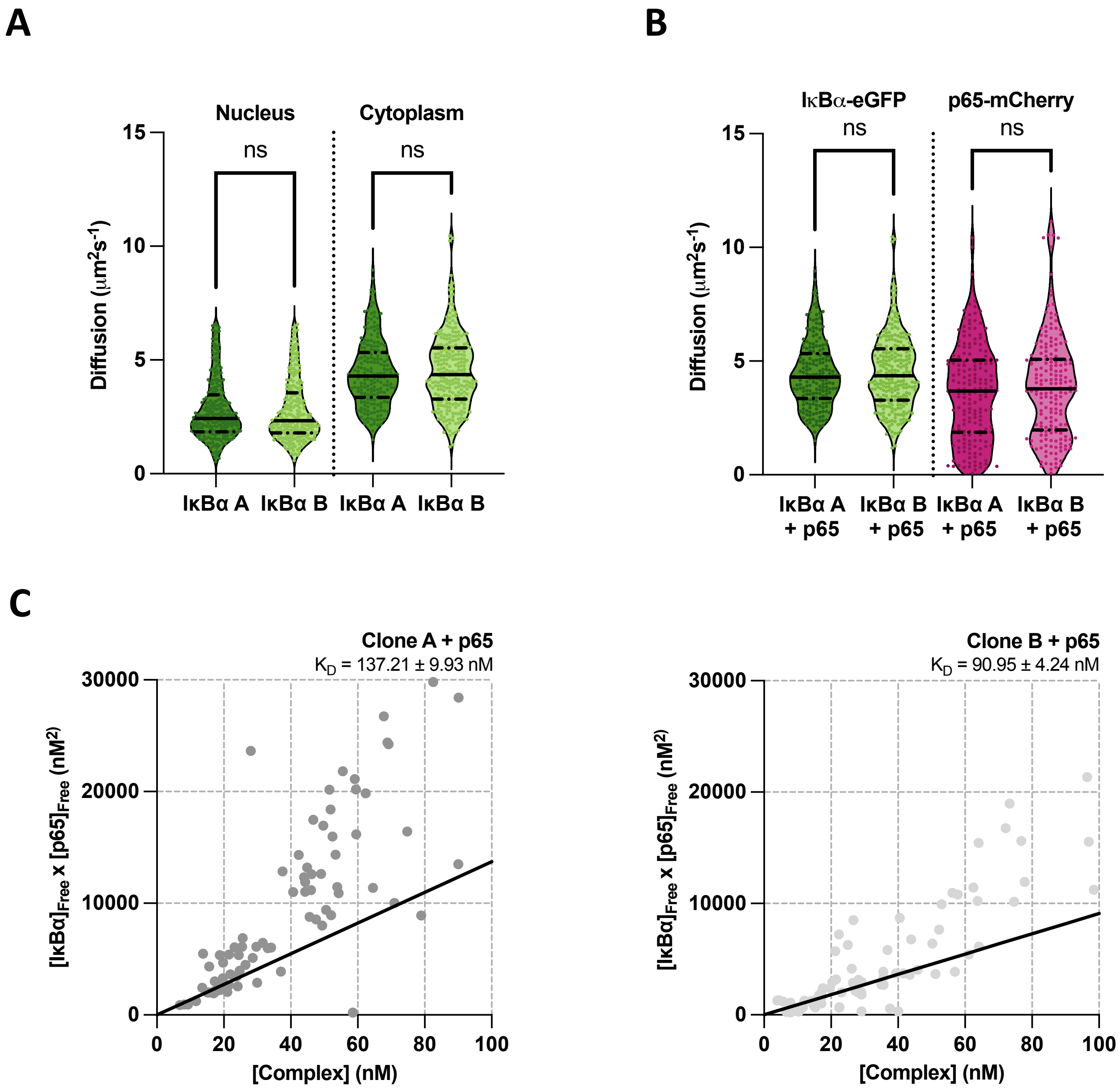

Supplementary Figure S1. Diffusion and interaction of IκBα-eGFP and p65-mCherry.

A: Measurement of diffusion of IκBα-eGFP in the nucleus and cytoplasm of clone A and clone B cells. Median and interquartile range are indicated on each distribution. Kruskal-Wallis test, Dunn's multiple comparison correction.

B: Measurement of diffusion of IκBα-eGFP and p65-mCherry in the cytoplasm of clone A + p65 and clone B + p65 cells. Median and interquartile range are indicated. Kruskal-Wallis test, Dunn's multiple comparison correction.

C: Determination of interaction strength between IκBα and p65 in the cytoplasm of clone A + p65 (left) clone B + p65 (right) cells. Quantile regression was used to calculate the K_D and error.

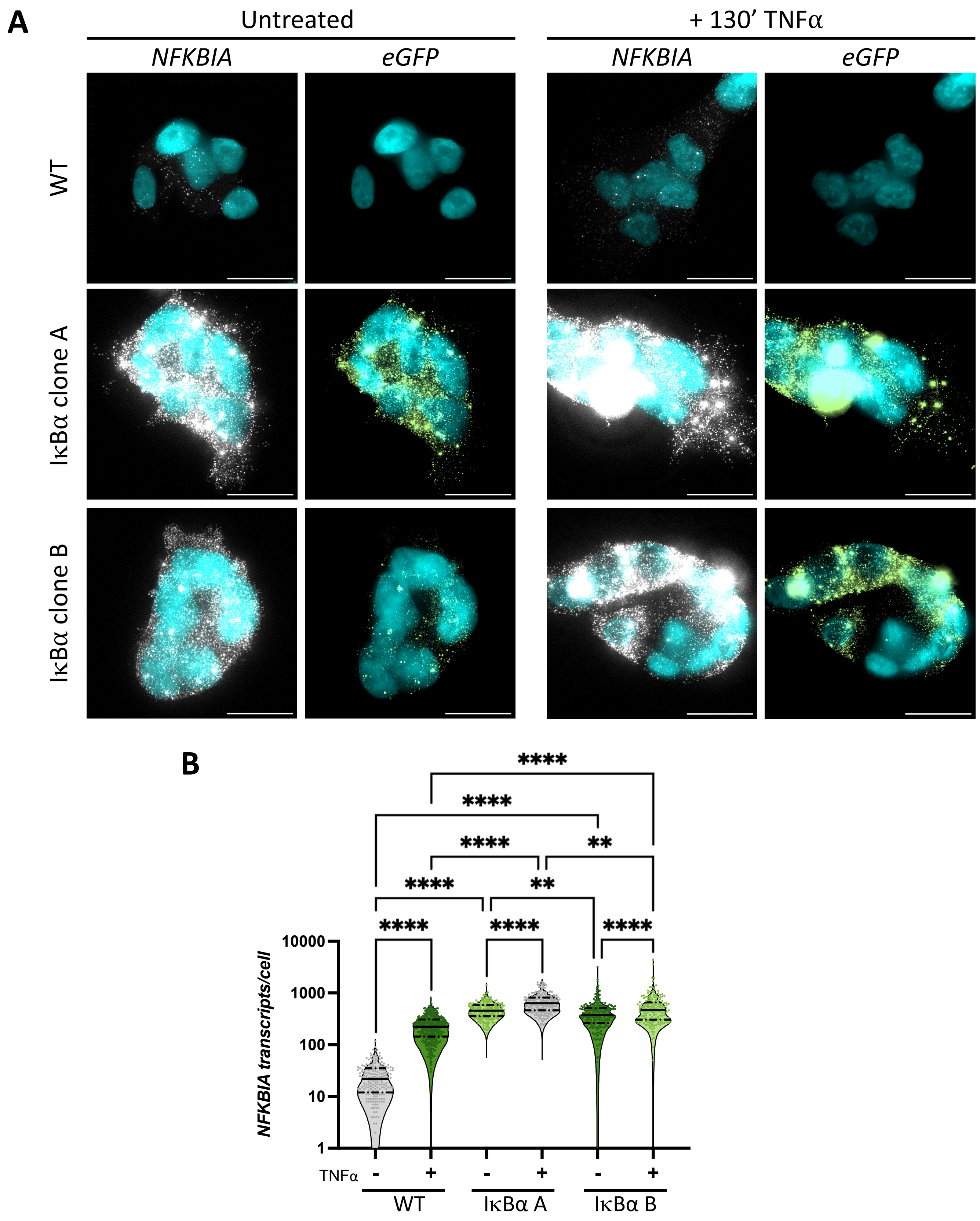

Supplementary Figure S2. smRNA-FISH analysis of cell line response to TNFα treatment.
A: smRNA-FISH detection of *NFKBIA* and *eGFP* transcripts with and without 130' TNFα treatment. Probe fluorescence is shown in white and green; DAPI counterstaining of nuclei is shown in cyan. Display settings are standardised for each fluorescence channel, resulting in signal saturation at bright spots. Scale bars = 20 μm.
B: Quantification of *NFKBIA* by smRNA-FISH detection in clonal cell lines with and without TNFα treatment. N = 200-400 cells/condition, imaged over 2-3 independent experiments. Two-way ANOVA, Tukey's multiple comparison correction.

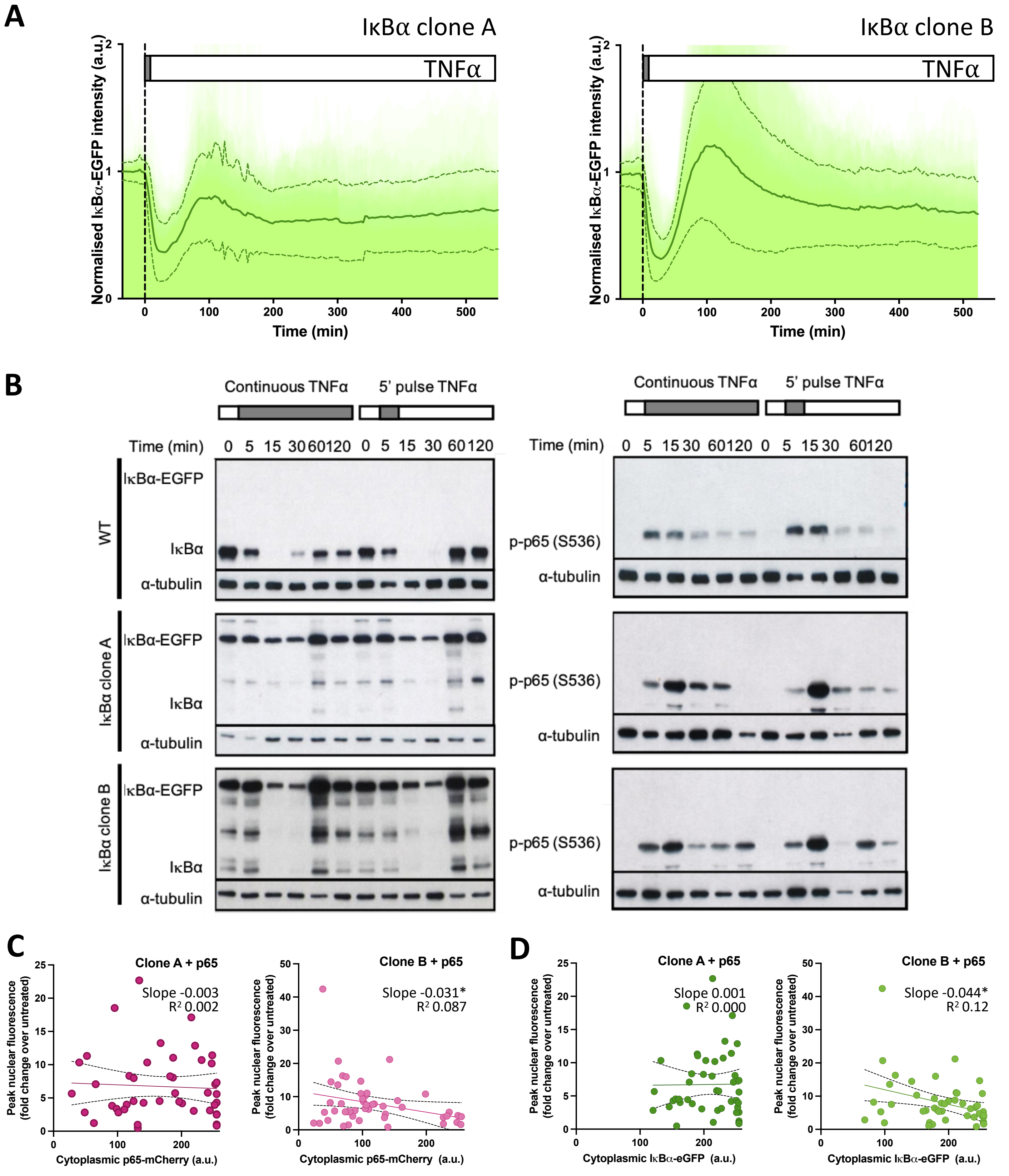

Supplementary Figure S3. Dynamic response of IκBα-eGFP reporter cells.

A: Clonal cell response to a 5 minute pulse of TNFα treatment. Individual traces are shown in pale green; population average +/- SD is shown in dark green. n = 140-150 cells imaged over at least 6 independent experiments.

B: Western blot analysis of protein response to TNFα treatment.

C: Comparison of peak p65-mCherry fluorescence to basal cytoplasmic p65-mCherry level. N = 48 cells. * indicates significant difference of linear regression slope from zero.

D: Comparison of peak p65-mCherry fluorescence to basal cytoplasmic IκBα-eGFP level. N = 45 cells. * indicates significant difference of linear regression slope from zero.

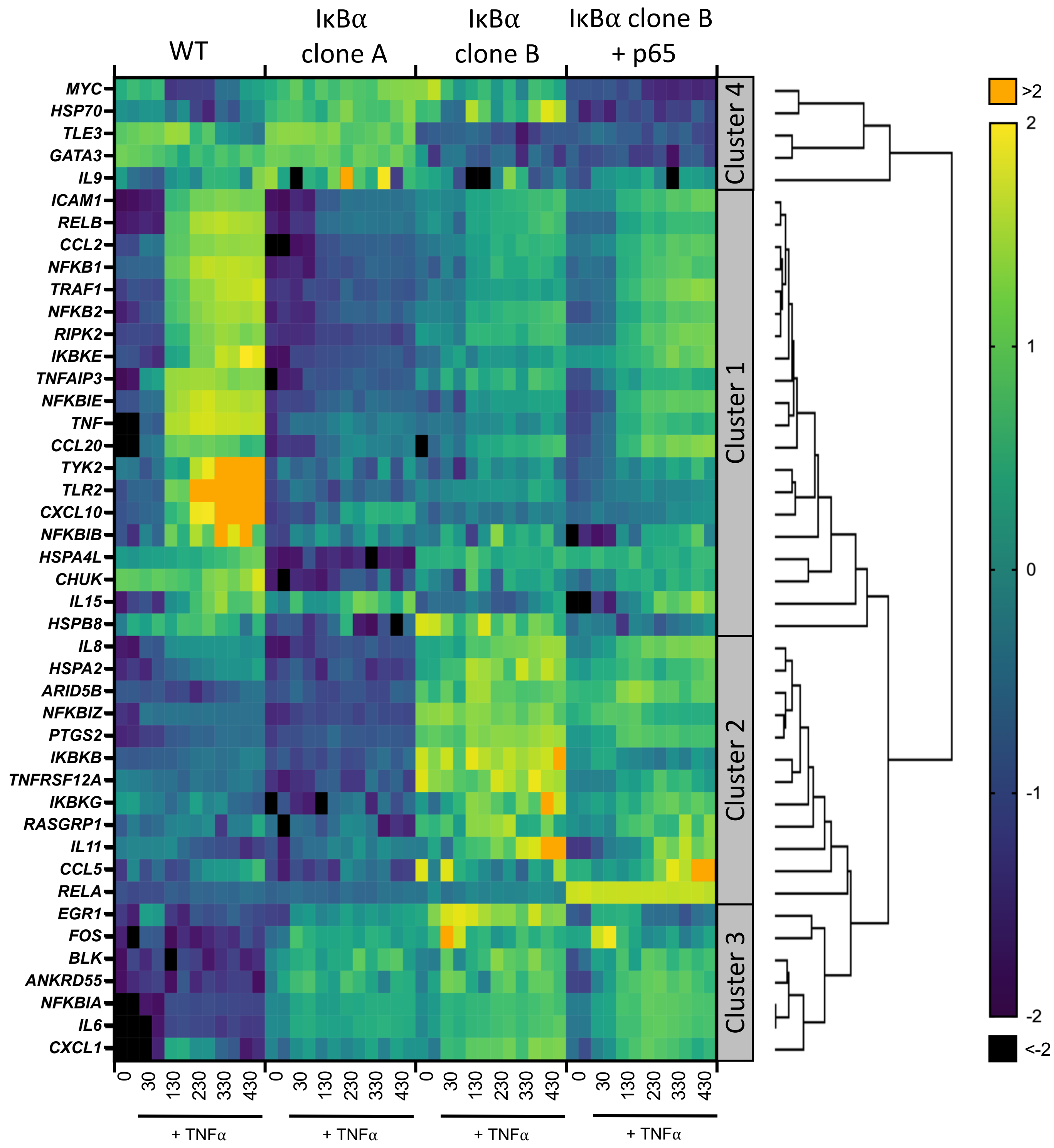

Supplementary Figure S4. Nanostring analysis of cell line gene expression. Heatmap representation of gene expression of clonal cell lines in response to TNFα treatment, as determined by Nanostring gene expression assay. N = 2/time/cell line. Expression is normalised to five housekeeping genes and internal control probes, then scaled to average gene expression level.

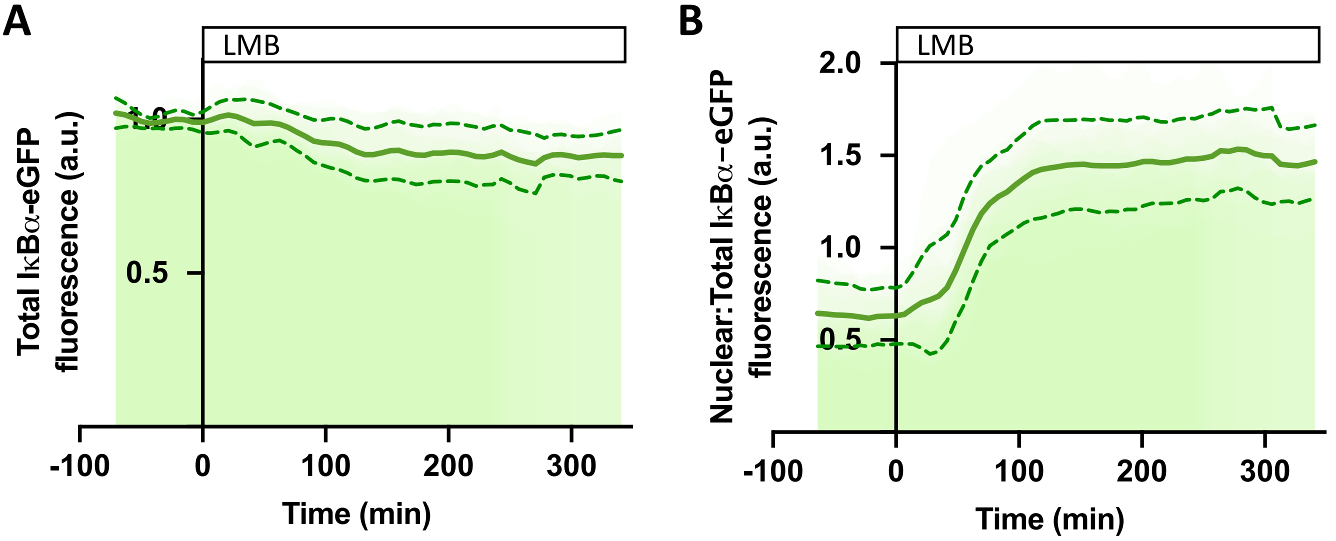

Supplementary Figure S5. Quantification of photobleaching over experimental time series.
A: Change in total IκBα-eGFP fluorescence over LMB treatment time course experiments.
B: Change in nuclear:total IκBα-eGFP fluorescence ratio over LMB treatment time course experiments. Individual traces are shown in pale green; population average +/- SD is shown in dark green. N = 15 cells.

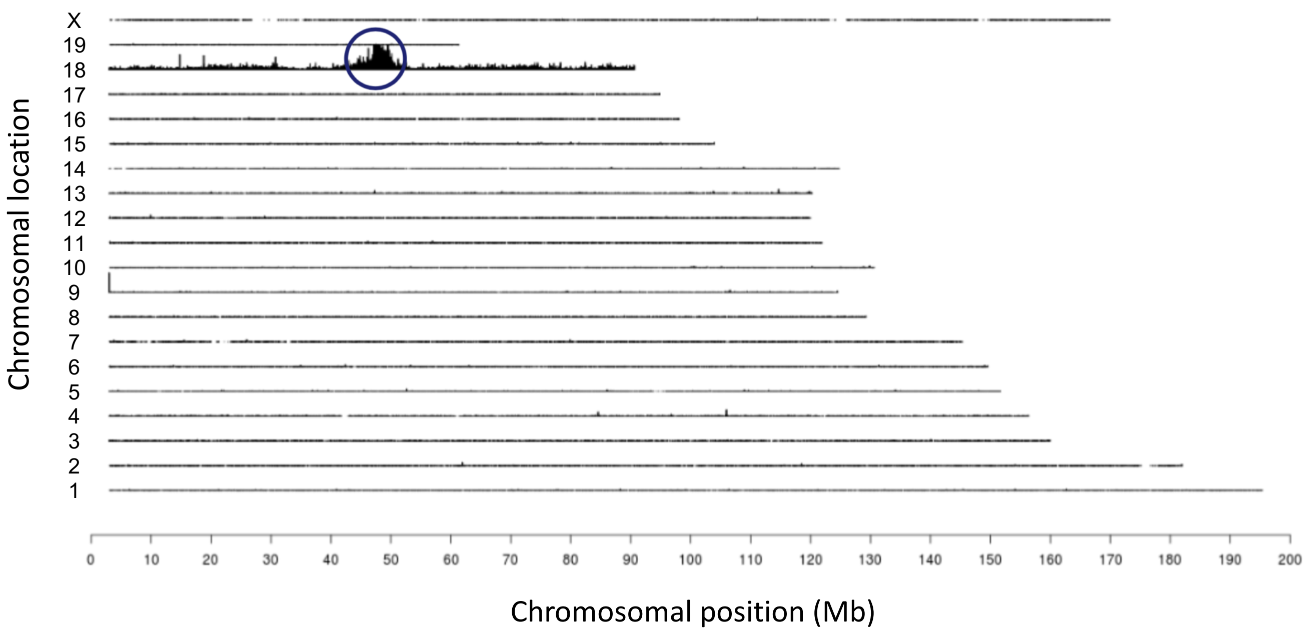

Supplementary Figure S6. Copy number analysis of transgene integration.
Targeted locus amplification and sequencing identified a single integration site of the IκBα-eGFP fusion BAC in mouse chromosome 18. Integration copy number was estimated as 2-6 copies based on read coverage.

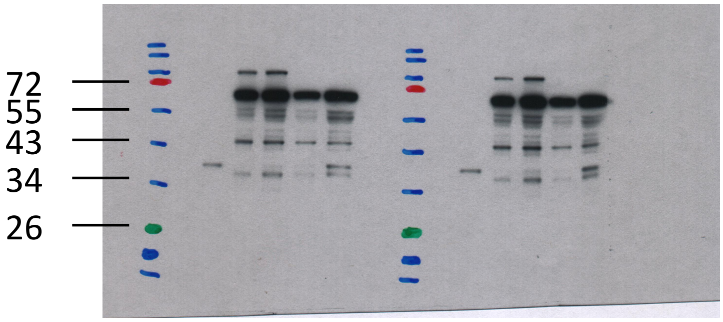

Figure 1E
IκBα

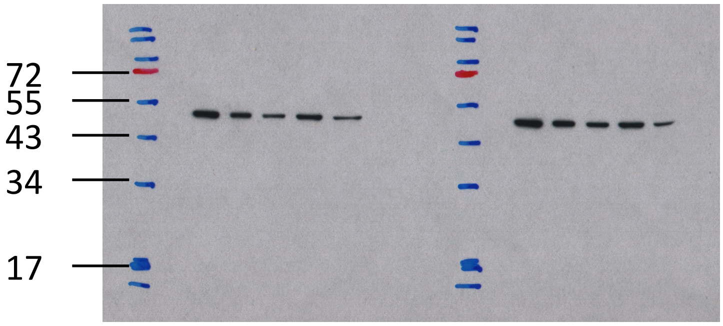

Figure 1E
α-tubulin

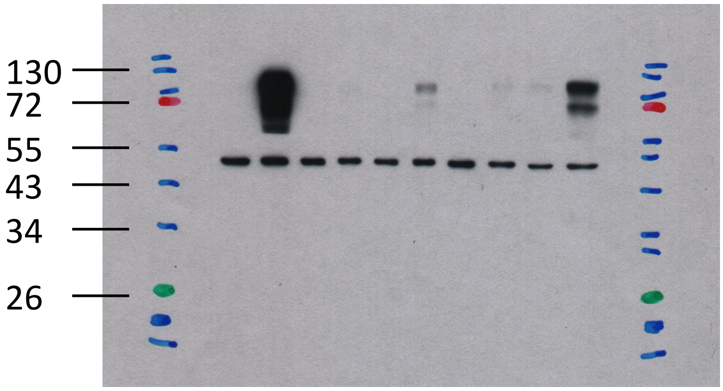

Figure 3E
ICAM1 (top)
α-tubulin (bottom)

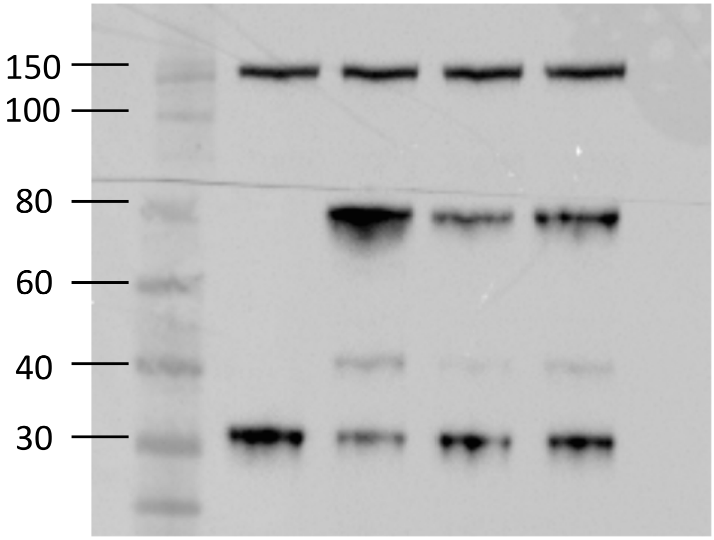

Figure 5B
Vinculin (top)
IκBα (bottom)

Supplementary Figure S7. Unprocessed Western blot images.
Protein ladder used: NEB P7712S (Figure 1E and 3E) or Thermo Scientific 84785 (Figure 5B).
